# Pale Body-Like Inclusion Formation and Neurodegeneration following Depletion of 26S Proteasomes in Mouse Brain Neurones are Independent of α-Synuclein

**DOI:** 10.1371/journal.pone.0054711

**Published:** 2013-01-30

**Authors:** Simon M. L. Paine, Glenn Anderson, Karen Bedford, Karen Lawler, R. John Mayer, James Lowe, Lynn Bedford

**Affiliations:** 1 Neural Development Unit, University College London Institute of Child Health, London, United Kingdom; 2 Department of Histopathology, Great Ormond Street Hospital for Children NHS Trust, London, United Kingdom; 3 Hull and East Yorkshire Hospitals, Hull Royal Infirmary, Hull, United Kingdom; 4 School of Biomedical Sciences, University of Nottingham, Nottingham, United Kingdom; 5 Division of Histopathology, School of Molecular Medical Sciences, University of Nottingham, Nottingham, United Kingdom; Johns Hopkins, United States of America

## Abstract

Parkinson’s disease (PD) is characterized by the progressive degeneration of substantia nigra pars compacta (SNpc) dopaminergic neurones and the formation of Lewy bodies (LB) in a proportion of the remaining neurones. α-synuclein is the main component of LB, but the pathological mechanisms that lead to neurodegeneration associated with LB formation remain unclear. Three pivotal elements have emerged in the development of PD: α-synuclein, mitochondria and protein degradation systems. We previously reported a unique model, created by conditional genetic depletion of 26S proteasomes in the SNpc of mice, which mechanistically links these three elements with the neuropathology of PD: progressive neurodegeneration and intraneuronal inclusion formation. Using this model, we tested the hypothesis that α-synuclein was essential for the formation of inclusions and neurodegeneration caused by 26S proteasomal depletion. We found that both of these processes were independent of α-synuclein. This provides an important insight into the relationship between the proteasome, α-synuclein, inclusion formation and neurodegeneration. We also show that the autophagy-lysosomal pathway is not activated in 26S proteasome-depleted neurones. This leads us to suggest that the paranuclear accumulation of mitochondria in inclusions in our model may reflect a role for the ubiquitin proteasome system in mitochondrial homeostasis and that neurodegeneration may be mediated through mitochondrial factors linked to inclusion biogenesis.

## Introduction

Parkinson’s disease (PD) is defined pathologically by the loss of dopaminergic (DA) neurones from the substantia nigra pars compacta (SNpc) and the accumulation of aggregated α-synuclein in Lewy bodies (LB) in a proportion of the remaining neurones. Attention is increasingly turning to the concept of proteostasis in diseases in which abnormal protein accumulation develops in association with the formation of inclusion bodies [1]. The formation of inclusions is likely to be cytoprotective [2], with neuronal death being the result of failure to compensate. Therefore, it is important to understand which cellular systems are involved in the formation of inclusion bodies and what leads to neuronal death in this context. Three elements are regarded as pivotal in the pathogenesis of neurodegeneration in PD: α-synuclein, mitochondria and protein degradation systems.

As well as being the defining protein component of LB [3], point mutations and multiplication of the *Snca* locus, encoding α-synuclein, lead to familial forms of the disease, and *Snca* variation is associated with an increased risk of developing sporadic PD [4], [5]. LB are a feature of these forms of PD and are present in a wide range of neuronal populations beyond the mesencephalon. Their presence in the neocortex and limbic system is seen in the second most common form of dementia, dementia with Lewy bodies (DLB) [6], [7]. LB pathology often coexists with other neurodegenerative disorders, such as Alzheimer’s disease (AD) [8]. Despite 15 years of intense study since *Snca* was first linked to PD [3], the pathological mechanisms that lead to neurodegeneration associated with LB formation remain unclear.

Extensive data implicates mitochondrial factors in neurodegeneration [9], [10]. In addition to several genetic factors known to cause familial PD linking to mitochondria (e.g. Parkin), respiratory chain defects in the SN, and other tissues, from sporadic PD cases have long been recognized and neurotoxins that act via various mechanisms to impair mitochondrial complex I have been used to model the disease [9]–[11]. Mitochondria are a prominent component of pale bodies (PB), the precursor of LB [12], [13].

Ubiquitin is key to the two main cellular degradation systems: the ubiquitin proteasome system (UPS) and the autophagy-lysosomal pathway (ALP) [14]. Ubiquitin-positive inclusions are a consistent feature of the majority of human neurodegenerative diseases, suggesting involvement of protein degradation systems [15]–[17]. The UPS and ALP have specific roles in the degradation of α-synuclein *in vivo*; under normal conditions the UPS is the main degradation system for α-synuclein whilst the ALP is recruited with raised α-synuclein levels [18]. Also relevant is that the formation of LB has been regarded as an aggresomal process, involving components of the UPS as well as the ALP [19], [20].

Recent attention has focused on the importance of protein degradation systems in relation to mitochondrial function [21]. The UPS has a role in mitochondrial protein quality control [22], [23], and specifically, mitophagy (the selective degradation of dysfunctional mitochondria by autophagy). Aberrant mitochondria are selected for mitophagy by PINK1-dependent recruitment of cytosolic Parkin, an E3 ubiquitin ligase implicated in PD, which mediates K48- and K63-linked polyubiquitination associated with the UPS and ALP respectively [24], [25]. There is, therefore, a clear point of convergence between two elements known to be important in PD pathogenesis: mitochondria and protein degradation systems. Interestingly, α-synuclein has also recently been found to be directly involved in mitochondrial homeostasis [26]–[28].

We previously reported that 26S proteasomal depletion in mouse brain neurones, including SNpc DA neurones, causes neurodegeneration and the formation of Lewy-like inclusions resembling human PB [13]. The mouse inclusions are defined as PB-like because they are morphologically, neuropathologically and ultrastructurally similar to PB found in human disease, the precursor of LB. They contain endogenously formed fibrillar α-synuclein, ubiquitin, membranous material in vesicles and mitochondria. Although still a model system, it is important to emphasize that this is an *in vivo* mammalian model that brings together several of the pivotal elements implicated in sporadic PD, namely: a defect in the UPS, α-synuclein aggregation and mitochondria. Therefore, this is a model in which to explore fundamental biological connections between these elements in the context of inclusion formation and neuronal death.

Using this model, we tested the hypothesis that α-synuclein is essential for the neurodegeneration and PB-like inclusion formation that characterize PD. We show here that the ability of 26S proteasomal impairment to cause neuronal death and inclusion formation in the SNpc is not moderated by the absence of α-synuclein. Significantly, this was not a specific response limited to mesencephelic DA cells; α-synuclein does not modify processes leading to cortical PB-like inclusions. We also demonstrate that the ALP is not activated in 26S proteasome-depleted mouse brain neurones.

## Materials and Methods

### Ethics Statement

All procedures were carried out under personal and project licenses granted by the UK Home Office in accordance with the Animals (Scientific Procedures) Act 1986 and with ethical approval from the University of Nottingham Ethical Review Committee. Animal welfare was ensured and no steps were necessary to ameliorate suffering. Animals were perfusion-fixed or sacrificed using an approved S1 method.

### Animals

Mice lacking the first two exons and upstream untranslated region of the α-synuclein gene (*Snca*), encoding amino acids 1–41 of α-synuclein, were kindly provided by Prof. Vladimir Buchman, University of Cardiff, Wales UK [29]. These mice (*Snca* ^−/−^) were crossed with floxed *Psmc1* mice (*Psmc1* ^fl/fl^) expressing Cre recombinase from the tyrosine hydroxylase (*TH*) promoter (*TH* ^Cre^) to achieve catecholaminergic neurone-specific inactivation of *Psmc1* (*Psmc1* ^fl/fl^;*TH* ^Cre^) [13]. TH is expressed from mid-gestation in midbrain dopaminergic neurones [30]. For forebrain neurone-specific inactivation of *Psmc1*, Cre recombinase was expressed under the control of the calcium calmodulin-dependent protein kinase IIα promoter (*Psmc1* ^fl/fl^;*CaMKIIα-Cre*) [13]. CaMKIIα is expressed in post-mitotic neurones from approximately post-natal week 2 [31], [32]. All mice were male. Appropriate littermate mice were used as controls.

### Genotyping

Genomic DNA was used in a polymerase chain reaction, 62°C annealing. Primers: *Cre* (sense) GGTAGCACCGCAGGTGTAG; *Cre* (antisense) CTAATCGCCATCTTCCAGCAG; *Psmc1* (wild-type and floxed sense) TACGAACCTCCTGTCCCAAC; *Psmc1* (knock-out sense) CAGAAATACAGCCAGTGACC; *Psmc1* (common antisense) CTGGAACTCAGTGGATTGAG; *Snca* (common sense) CAGCTCAAGTTCAGCCACGA; *Snca* (wild-type antisense) AAGGAAAGCCGAGTGATGTAC; *Snca* (knock-out antisense) ATGGAAGGATTGGAGCTACG.

### Histology

For light microscopy, mice were perfused with 0.9% saline followed by 4% paraformaldehyde (PFA) in phosphate buffered saline pH 7.4. PFA-fixed brains were processed to paraffin with chloroform as the clearing agent. General morphological examination used haematoxylin and eosin staining. Immunostaining was performed as directed in Vector Laboratories M.O.M Immunodetection or Vectastain Elite Rabbit IgG ABC kits. Antigen retrieval used 0.01 M citrate buffer containing 0.05% Tween-20 pH 6. Primary antibody incubation was for 1 hour at room temperature [tyrosine hydroxylase (Sigma), α-synuclein (BD Biosciences), p62 (Enzo Life Sciences), ubiquitin K63-isopeptide motif (Enzo Life Sciences), lysine 48-linkage specific polyubiquitin (Millipore), in-house ubiquitin, COX IV (Cell Signalling), cytochrome C (Santa Cruz), LC3B (Sigma) and Cathepsin D (Cell Signaling and Abcam)]. Double immunofluorescent staining used similar methods; primary (from different species) and appropriate fluorescent secondary antibodies were incubated in sequence. For electron microscopy, mice were perfused with 0.9% saline followed by gluteraldehyde fixative suitable for EM.

### Stereology

Horizontal sections were cut at 7 micrometres. Two consecutive sections were collected onto a single APES-coated slide starting rostral to the substantia nigra and continuing through to the brainstem. Every seventh slide through the SNpc was stained for TH and counterstained with haematoxylin [33]. The slides were scanned and analysed with ImageJ. The SNpc was delineated using anatomical landmarks and neuronal cytology. Neurones were counted if they fell within the outline of the SNpc, possessed the characteristic cytological features of a neurone within the SNpc, had clear cytoplasmic TH immunoreactivity and the dominant nucleolus was present only in the reference (lower) section. The count was repeated three times in each of three animals for each genotype. The sum of the neurones counted for each SNpc was divided by the sampling interval in order to produce an estimate of the total number of neurones for each animal. The same stereological methods, using haematoxylin and eosin stained sections, were applied to estimate the total number of intraneuronal inclusions within the SNpc of the same animals. Estimation of reference volume used Cavalieri. Statistical significance was assessed using analysis of variance (ANOVA) and Tukey HSD *post hoc* testing.

### Western Blotting

Tissue was homogenized in cold lysis buffer [50 mM Tris-HCl pH 7.4, 150 mM NaCl, 1 mM EDTA, 1% Triton X-100; 0.1% SDS, 0.5% sodium deoxycholate, protease inhibitors], incubated on ice for 30 minutes, centrifuged at 16000×g to eliminate cellular debris and protein quantified using the Bradford assay. Proteins were separated by SDS-PAGE and transferred to nitrocellulose membrane. Blocking used 5% Marvel in tris buffered saline-0.1% Tween-20. Incubation in primary [1∶2000 LC3B (Sigma); 1∶1000 Cathepsin D (Cell Signaling and Abcam) and β-actin (Sigma)] and appropriate horseradish peroxidise-conjugated secondary antibodies (Sigma) was for 1 hour, room temperature. Proteins were visualized with ECL substrate (Pierce). Quantity One 1-D analysis Software was used to calculate band intensity.

## Results

### α-synuclein Aggregation does not Modify Neurodegeneration and is not Essential for the Biogenesis of PB-like Inclusions in 26S Proteasome-depleted Neurones

We previously demonstrated impaired 26S proteasome function in mouse neurones, including SNpc DA neurones, caused neurodegeneration and the formation of intraneuronal PB-like inclusions [13]. Specifically 26S proteasomal dysfunction was achieved by Cre/loxP conditional genetic targeting of *Psmc1*, a key ATPase subunit of the 19S regulatory particle of the 26S proteasome. We showed PSMC1 was responsible for the assembly and activation of the 26S proteasome in mouse neurones *in vivo*; the 20S proteolytic core particle and therefore ubiquitin-independent proteasomal degradation was not affected [13]. To study whether α-synuclein directly contributes to the formation of inclusions and degeneration of DA neurones in the mouse SNpc, we generated mice constitutively lacking α-synuclein (*Snca* ^−/−^) and in which the 26S proteasome was impaired in TH-positive neurones (*Psmc1* ^fl/fl^;*TH* ^Cre^).

**Figure 1:**
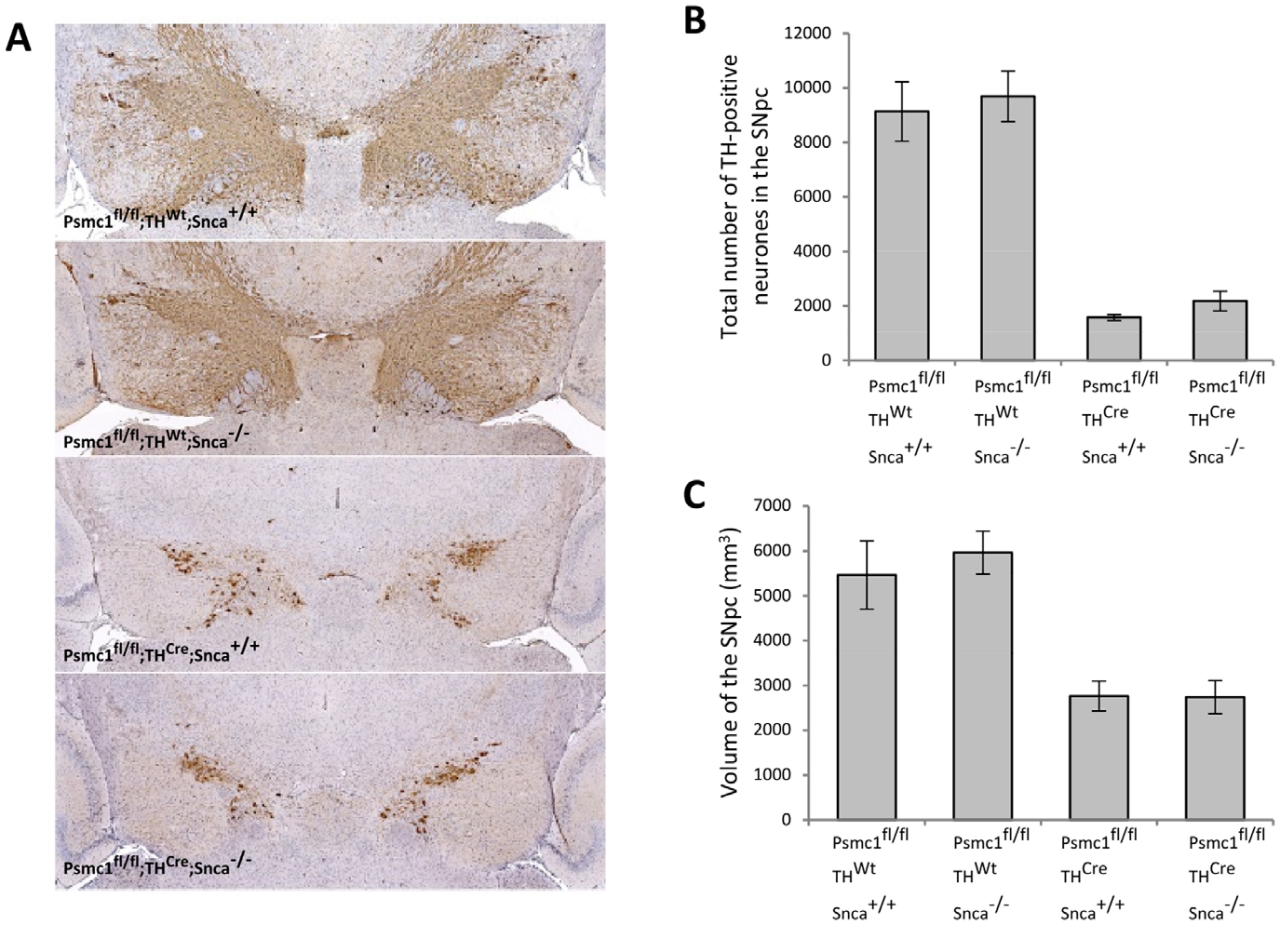
Neurodegeneration of 26S proteasome-depleted SNpc neurones is not modified by the absence of α-synuclein. (A) TH-immunoreactive neurones at the level of the SNpc. 5×. (B) The total number of TH-positive neurones within the SNpc. n = 3, P<0.01 (ANOVA, Tukey HSD post hoc test). (C) Volume of the SNpc containing TH-positive neurones. n = 3, P<0.01 (ANOVA, Tukey HSD post hoc test). Control (*Psmc1* ^fl/fl^;*TH* ^Wt^) *vs.* 26S proteasome-depleted (*Psmc1* ^fl/fl^;*TH* ^Cre^); presence (*Snca* ^+/+^) *vs.* absence (*Snca* ^−/−^) of α-synuclein. Error bars indicate SEM.

Immunostaining of the mouse SNpc showed a severe loss of TH-positive neurones in *Psmc1* ^fl/fl^;*TH* ^Cre^ mice (Figure 1A). Our original publication of this model demonstrates that this is neurodegeneration and not decreased TH expression by TH and Nissl staining of adjacent sections from the SNpc. We show results from 3-week-old mice because autonomic dysfunction, as a consequence of 26S proteasomal depletion in all catecholaminergic (TH) neurones, causes premature death [13]. We recognize that these are young mice, but emphasize that the neuropathology following 26S proteasomal dysfunction in SNpc neurones involves elements pivotal to PD pathways and therefore, this is an important *in vivo* model.

Quantification of the total number of TH-positive neurones showed that the 26S proteasome-depleted mice (*Psmc1* ^fl/fl^;*TH* ^Cre^) possessed approximately 85% fewer neurones when compared with control mice (*Psmc1* ^fl/fl^;*TH* ^Wt^), irrespective of the presence (*Snca* ^+/+^) or absence (*Snca* ^−/−^) of α-synuclein (Figure 1B). In keeping with the analysis of TH-positive neurone number, there was a significant reduction in the volume of the SNpc when either group of control mice (*Psmc1* ^fl/fl^;*TH* ^Wt^) was compared with either group of 26S proteasome-depleted mice (*Psmc1* ^fl/fl^;*TH* ^Cre^) (Figure 1C). That is, the absence of α-synuclein (*Snca* ^−/−^) had no significant effect on the volume of the SNpc.

**Figure 2:**
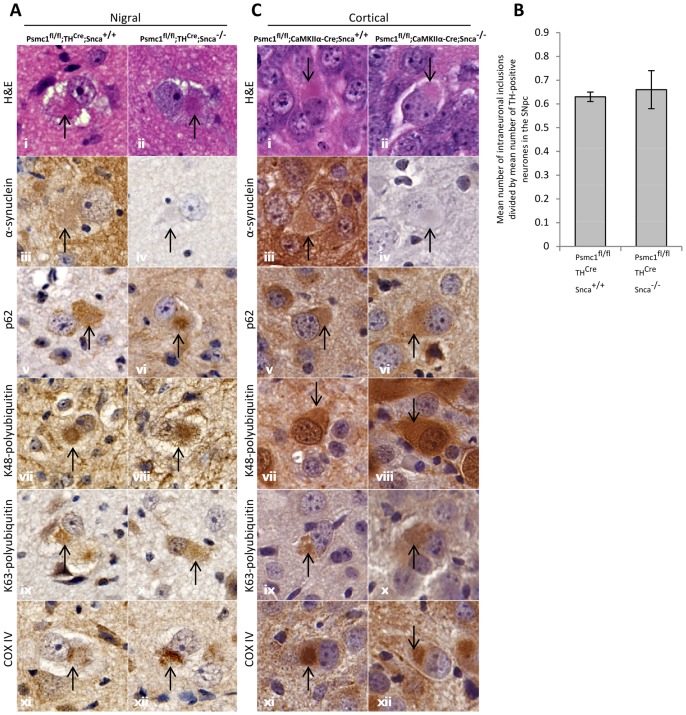
The absence of α-synuclein does not modify PB-like inclusion biogenesis or morphology following 26S proteasomal depletion in mouse brain neurones. (A and C) Representative inclusions (arrows) in neurones of the SNpc (*Psmc1* ^fl/fl^;*TH* ^Cre^, A) and cortex (*Psmc1* ^fl/fl^;*CaMKIIα-Cre*, C) in the presence (*Snca* ^+/+^) and absence (*Snca* ^−/−^) of α-synuclein; from the top: hematoxylin and eosin staining, α-synuclein, p62, K48- and K63-specific polyubiquitin, and COX IV. 63×. (B) Absence of α-synuclein (*Snca* ^−/−^) has no effect on the proportion of TH-positive neurones bearing eosinophilic inclusions in the 26S proteasome-depleted (*Psmc1* ^fl/fl^;*TH* ^Cre^) SNpc. n = 3, no significant difference (ANOVA). Error bars indicate SEM.

We previously demonstrated the formation of intraneuronal eosinophilic PB-like inclusions following 26S proteasomal depletion in surviving mouse SNpc neurones (*Psmc1* ^fl/fl^;*TH* ^Cre^;*Snca* ^+/+^), exhibiting α-synuclein, ubiquitin, p62 and cytochrome oxidase IV (COX IV) immunoreactivity (Figure 2Ai,iii,v,vii,ix,xi) [13]. Careful morphological and immunophenotypical examination revealed, with the exception of α-synuclein (Fig. 2Aiv), that the inclusions in SNpc neurones lacking α-synuclein (*Psmc1* ^fl/fl^;*TH* ^Cre^;*Snca* ^−/−^) were similarly eosinophilic and immunoreactive to p62, ubiquitin and COX IV (Figure 2Aii,vi,viii,x,xii). We sought to quantify whether the number of PB-like inclusions in TH-positive neurones resulting from loss of 26S proteasomes was modified by α-synuclein. The total number of eosinophilic inclusions in the SNpc was counted and divided by the total number of neurones present in this region with the potential to form inclusions, i.e. TH-positive neurones. Figure 2B shows that the proportion of neurones bearing inclusions was similar in both groups of 26S proteasome-depleted mice, irrespective of the presence (*Psmc1* ^fl/fl^;*TH* ^Cre^;*Snca* ^+/+^) or absence (*Psmc1* ^fl/fl^;*TH* ^Cre^;*Snca* ^−/−^) of α-synuclein.

In order to assess whether this was a specific response of mesencephelic DA cells, we conditionally deleted *Psmc1* in mouse forebrain neurones by expressing Cre recombinase from the *calcium calmodulin-dependent protein kinase IIα* promoter (*CaMKIIα-Cre*), causing PB-like inclusions in cortical neurones (*Psmc1* ^fl/fl^;*CaMKIIα-Cre*) [13]. Significantly, we found here too the absence of α-synuclein (*Snca* ^−/−^) does not modify the processes leading to cortical PB-like inclusion formation (Figure 2C). Intranuclear inclusions were not identified in any of the mice.

### Accumulation of Aberrant Mitochondria

**Figure 3:**
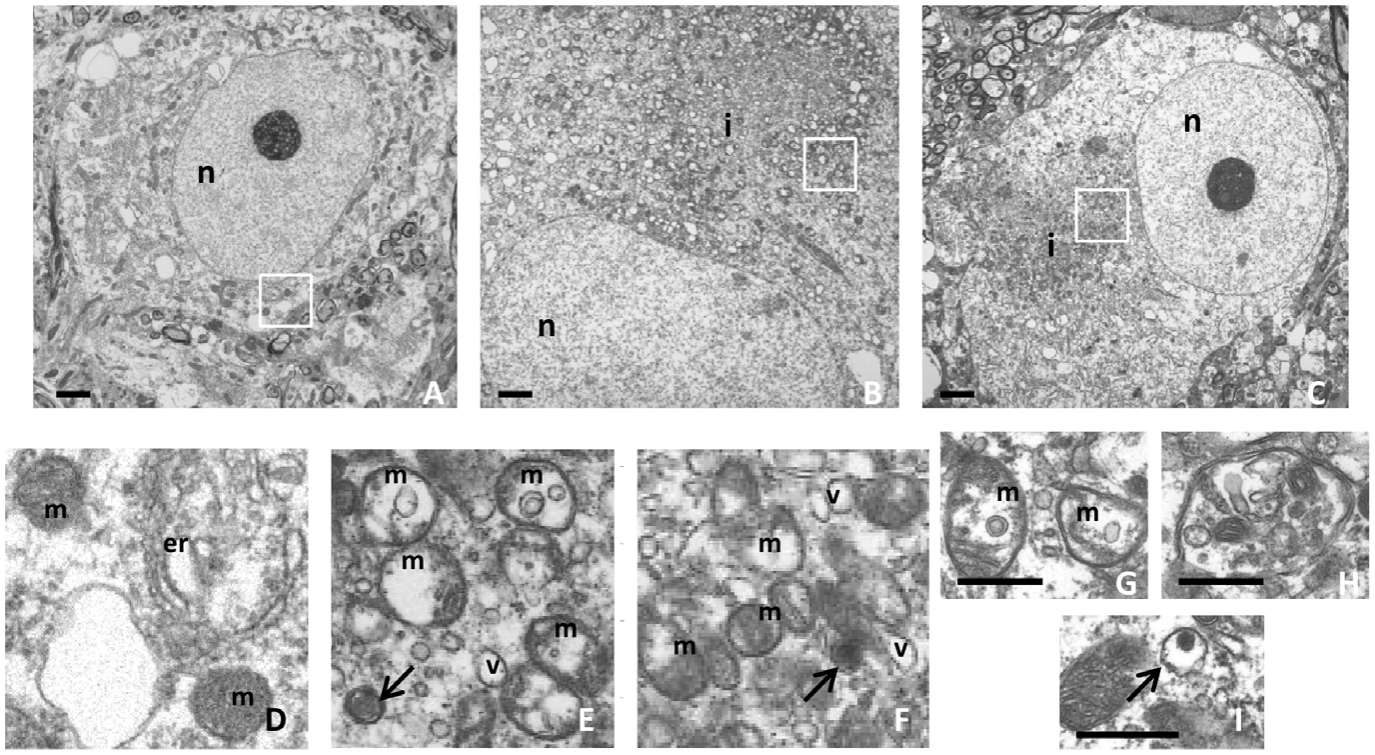
The absence of α-synuclein does not modify the ultrastructure of PB-like inclusions in mouse SNpc neurones. Representative electron micrographs of control (A; *Psmc1* ^fl/fl^;*TH* ^Wt^) and 26S proteasome-depleted SNpc neurones with (B; *Psmc1* ^fl/fl^;*TH* ^Cre^;*Snca* ^+/+^) and without (C; *Psmc1* ^fl/fl^;*TH* ^Cre^;*Snca* ^−/−^) α-synuclein. Enlarged views of the boxed areas are shown in D-F respectively. The inclusions contain mainly abnormal mitochondria (E-G; **m**) interspersed with numerous small vesicles (E and F; **v**). Autophagosome-like structures containing electron-dense material (E, F and I; arrows) as well as recognizable cytosolic elements including mitochondria (H) are present. **n**, nucleus; **i**, PB-like inclusion; **m**, mitochondria; **v**, vesicle and **er**, endoplasmic reticulum. Scale bar, 500 nm. *Psmc1* ^fl/fl^;*TH* ^Cre^;*Snca* ^+/+^ (G and H); *Psmc1* ^fl/fl^;*TH* ^Cre^;*Snca* ^−/−^ (I).

We used ultrastructural analysis to investigate more subtle pathological differences between PB-like inclusions in 26S proteasome-depleted mice with (*Psmc1* ^fl/fl^;*TH* ^Cre^;*Snca* ^+/+^) and without (*Psmc1* ^fl/fl^;*TH* ^Cre^;*Snca* ^−/−^) α-synuclein. This revealed that the intraneuronal inclusions in SNpc neurones were indistinguishable (Figure 3). The inclusions were composed predominantly of morphologically abnormal mitochondria, which, in general, had disrupted or disintegrated cristae (Figures 3D–G). The mitochondria within the inclusions were interspersed with membrane in numerous small vesicles and granular material (Figures 3E and 3F). We observed the presence of occasional autophagosome-like structures containing electron-dense material (Figure 3I) as well as recognizable cytoplasmic elements, including mitochondria (Figure 3H), but the majority of the vesicles were empty.

### The ALP is not Activated in 26S Proteasome-depleted Neurones

**Figure 4:**
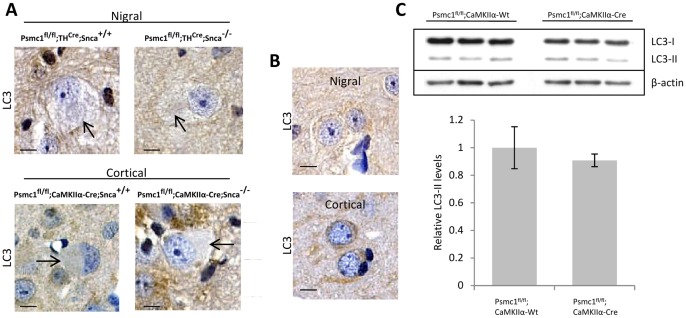
Autophagy is not activated in 26S proteasome-depleted mouse neurones. (A) Absence of LC3 immunoreactivity in representative neurones of the SNpc (*Psmc1* ^fl/fl^;*TH* ^Cre^, nigral) and cortex (*Psmc1* ^fl/fl^;*CaMKIIα-Cre*, cortical) in the presence (*Snca* ^+/+^) and absence (*Snca* ^−/−^) of α-synuclein. The arrows indicate PB-like inclusions. Scale bar, 10 µm. (B) The normal pattern of LC3 in nigral and cortical neurones shows a fine punctate cytoplasmic staining. Neurones from *Psmc1* ^fl/fl^;*TH* ^Wt^;*Snca* ^+/+^ (nigral) and *Psmc1* ^fl/fl^;*CaMKIIα-Wt*;*Snca* ^+/+^ (cortical) mice are shown, but the pattern of LC3 immunostaining was similar in the absence of α-synuclein. Scale bar, 10 µm. (C) Representative Western blot of LC3-I and LC3-II in total cortical homogenates from control (*Psmc1* ^fl/fl^;*CaMKIIα-Wt*) and 26S proteasome-depleted (*Psmc1* ^fl/fl^;*CaMKIIα-Cre*) mice. Graph depicts LC3-II levels normalized to β-actin. n = 4, no significant difference. Error bars indicate SEM.

Several studies have shown compensatory activation of the ALP with proteasome inhibition [18], [34], [35]. To investigate the involvement of the ALP following 26S proteasomal depletion in our model and whether this was associated with the membranous vesicles seen in inclusions, we carried out LC3 and cathepsin D immunostaining and Western blotting. LC3 is a marker of autophagosomes and post-translational modification of cytosolic LC3-I to LC3-II that is associated with autophagosomal membranes is an index of autophagy activation [36], [37]. Punctate endogenous LC3 immunostaining has also been associated with LC3-II and activation of autophagy with proteasome inhibition [35]. Overall, we did not consistently detect LC3-positive inclusions or the formation of LC3 puncta in 26S proteasome-depleted neurones, irrespective of the presence or absence of α-synuclein (Figures 4A and 4B). Further, LC3-cytochrome C double-immunofluorescent staining did not demonstrate co-localization of LC3 and mitochondria in inclusions following 26S proteasomal depletion, but LC3 staining was intermixed with dispersed mitochondria in both 26S proteasome-depleted and control neurones (Figure S1A). We report that variable LC3 staining was evident in some inclusion bodies and is shown in Figure S1B. Interestingly, a recent study of Parkin-mediated mitophagy showed LC3 was associated with dispersed mitochondria, but absent from paranuclear mitochondrial aggregates, and that 26S proteasome function may facilitate their dispersal and uptake by the autophagic machinery [38].

To further evaluate the activation of autophagy, we measured the levels of LC3-II (ratio with β-actin) following Western blotting. Due to the small size and accuracy of reproducibly dissecting specifically the mouse SNpc, we used cortical tissue from control (*Psmc1* ^fl/fl^;*CaMKIIα-Wt*) and 26S proteasome-depleted mice (*Psmc1* ^fl/fl^;*CaMKIIα-Cre*). Supporting our immunohistochemical observations, the levels of LC3-II were not significantly different between 26S proteasome-depleted and control mice (Figure 4C). The levels of p62, a substrate of macroautophagy [39], were also similar (data not shown). However, the presence of p62 in inclusion bodies in 26S proteasome-depleted neurones is consistent with previous studies demonstrating it is required for aggregation of polyubiquitinated proteins and mitochondria [40], [41].

**Figure 5:**
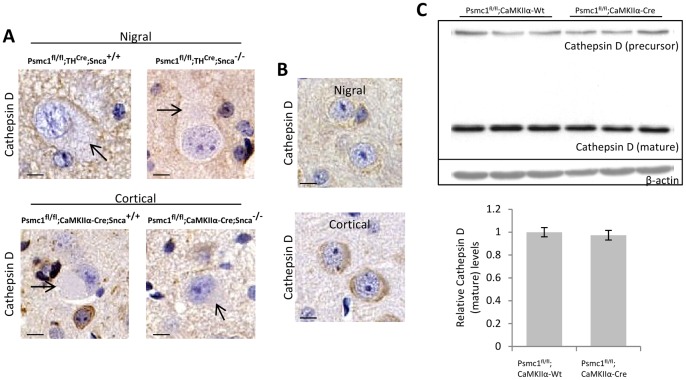
The lysosomal pathway is not activated in 26S proteasome-depleted neurones. (A) Representative immunostaining for cathepsin D in neurones of the SNpc (*Psmc1* ^fl/fl^;*TH* ^Cre^, nigral) and cortex (*Psmc1* ^fl/fl^;*CaMKIIα-Cre*, cortical) in the presence (*Snca* ^+/+^) and absence (*Snca* ^−/−^) of α-synuclein. Scale bar, 10 µm. The arrows indicate PB-like inclusions. (B) The normal pattern of cathepsin D in nigral and cortical neurones shows a fine punctate cytoplasmic staining. Neurones from *Psmc1* ^fl/fl^;*TH* ^Wt^;*Snca* ^+/+^ (nigral) and *Psmc1* ^fl/fl^;*CaMKIIα-Wt*;*Snca* ^+/+^ (cortical) mice are shown, but cathepsin D staining was similar in the absence of α-synuclein. Scale bar, 10 µm. (C) Representative Western blot and densitometric analysis of mature cathepsin D in total cortical homogenates from control (*Psmc1* ^fl/fl^;*CaMKIIα-Wt*) and 26S proteasome-depleted (*Psmc1* ^fl/fl^;*CaMKIIα-Cre*) mice. Graph depicts mature cathepsin D levels normalized to β-actin. n = 4, no significant difference. Error bars indicate SEM.

Following 26S proteasomal dysfunction we did not identify any inclusions in the SNpc or cortex that immunostained for cathepsin D, an essential lysosomal protease (Figures 5A and 5B). These observations are analogous to an *in vitro* study in cortical neurones modeling proteasomal dysfunction by applying pharmacological inhibitors that showed globular cathepsin D staining with activation of the ALP, but this was associated with dissolution of inclusions and did not co-localize with inclusion bodies [34]. Western blot and densitometric analysis showed the levels of mature cathepsin D were not significantly different between 26S proteasome-depleted and control mice (Figure 5C). Taken together, these results suggest that the ALP is not activated in 26S proteasome-depleted neurones.

## Discussion

In the present study we have investigated an *in vivo* model system of neurodegeneration and inclusion formation that brings together several of the pivotal elements implicated in PD. We have shown that α-synuclein is not essential for the biogenesis of PB-like inclusions and that it does not modify the neurodegeneration that follows *in vivo* depletion of 26S proteasomes in DA and cortical neurones. Additionally, we have shown that 26S proteasomal dysfunction does not lead to activation of the ALP.

In synucleinopathies such as PD and DLB, extensive research has focused on the over-expression of α-synuclein and its ability to aggregate [4], [5], [42]. Many transgenic mice have been created to over-express human PD variants of α-synuclein, but these models have not shown region-specific neuronal loss in the pattern seen in PD [4], [42]–[44]. The relevance of the 26S proteasomal depletion model to PD is still under debate because of conflicting data using 20S proteasomal inhibitors *in vivo* [45]–[50]. One of the strengths of our model system is that reproducible depletion of 26S proteasomes in mesencephelic DA neurones and non-catecholaminergic cortical neurones is achieved using conditional gene deletion, not the work of an inhibitor, which may have other effects [51]. However, we recognize that while the inclusions that form in 26S proteasome-depleted neurones have the characteristics of PB, we do not know if these would develop into LB if the neurones survived. Importantly, our work adds to the data on the pathological significance of the aggregated α-synuclein in neurodegeneration using a model in which α-synuclein itself is not over-expressed. We show that the 26S proteasomal depletion mouse model does not require α-synuclein aggregation for the manifestations of neurodegeneration.

*In vitro* and *in vivo* studies indicate that the proteasome is important for the degradation of α-synuclein, suggesting alterations in this pathway that lead to the abnormal expression or distribution of α-synuclein may contribute to α-synuclein neuropathology. In general, these studies have used pharmacological manipulation of the 20S proteasome that does not differentiate between ubiquitin-dependent and ubiquitin-independent degradation of the 26S and 20S proteasomes respectively [18], [52]–[54]. The 26S proteasome is a multi-protein complex composed of the 20S proteasome directly abutted by 19S regulatory particles [55]–[58]. Our model of 26S proteasomal depletion targets the 19S regulatory particle and does not affect 20S proteasome function, providing direct evidence that the 26S proteasome plays a role in α-synuclein degradation [13]. However, the present data suggests that 26S proteasomal depletion does not lead to pathological aggregation of α-synuclein. Since an early study showed ubiquitin-independent degradation of unmodified α-synuclein by purified 20S proteasomes *in vitro* [59], it is possible that the 20S proteasome plays a further role in α-synuclein degradation that would not be evident in our model and may be of pathological significance.

The relationship between α-synuclein and the proteasome is not straightforward. It has also been shown that aggregates of α-synuclein can impair 26S proteasome function, albeit at high concentrations [13], [18], [60]–[66]. It is conflicting, however, if α-synuclein can impair proteasome function, the absence of α-synuclein does not lead to less neurodegeneration in this study. Few studies have investigated proteasomal dysfunction by α-synuclein *in vivo* [18], [65] and our study suggests that the level of endogenous α-synuclein following 26S proteasomal depletion may not be sufficient itself to impair proteasome function.

Although α-synuclein is the gene arguably most closely linked to PD, multiple disease loci have been identified with relevance for familial and sporadic disease [67]. How each of these may lead to α-synuclein aggregation in LB is uncertain. Also, LB are present in other synucleinopathies as well as other neurodegenerative diseases, where, in general, α-synuclein is not known to be a primary causative factor, and a proportion of clinically normal people have LB, although this may be sub-threshold pathology [6]–[8], [68]–[70].

Our data provides evidence that the neurodegenerative processes that are initiated by, or lead to, proteasomal impairment may lead to the formation of α-synuclein inclusions. The paranuclear location of α-synuclein aggregation may be occurring as the consequence of activation of a cytoprotective pathway, e.g. an aggresome-like mechanism following proteasomal inhibition [19], and apart from α-synuclein, LB contain over 250 different proteins from diverse cellular processes [71]. However, in this context α-synuclein is not the downstream mediator of neurodegeneration because we show α-synuclein plays a redundant role in the formation of inclusion bodies and neurodegeneration. This has implications for all cases of LB in which there is no known mutation of the α-synuclein gene. We emphasize that our data does not exclude an important role for α-synuclein in PD and events that initiate neurodegeneration, but provides evidence that proteasomal dysfunction may be a relevant factor immediately responsible for neuronal death, which may be amplified by, as well as accompanied by, aggregation of α-synuclein.

Several *in vitro* studies, including primary cortical neurones, modeling proteasomal dysfunction by applying pharmacological inhibitors have shown 20S proteasome inhibition activates the ALP [34], [35]. Using similar methods to evaluate this pathway, we did not observe activation of the ALP in 26S proteasome-depleted mouse neurones *in vivo*. Our findings are consistent with a recent elegant *in vivo* study that only found cross-talk between the UPS and ALP in neurones with increased α-synuclein burden, i.e. in human wild-type α-synuclein transgenic mice. In normal mice, induction of the ALP was not observed following proteasome inhibition [18]. There are intrinsic differences between *in vitro* and *in vivo* experimental model systems as well as different species, but our findings extend the limited *in vivo* knowledge between the UPS and ALP degradative systems in SNpc and cortical neurones.

Although aggregation of undegraded proteins in inclusions may be an obvious non-specific consequence of disrupted neuronal homeostasis, the overt paranuclear accumulation of morphologically-impaired mitochondria in 26S proteasome-depleted neurones is significant. Models of proteasomal dysfunction using pharmacological 20S inhibitors have described neuronal death and the formation of cytoplasmic inclusions containing proteins that are present in LB, i.e. ubiquitin and α-synuclein, but not shown accumulation of mitochondria [34], [35]. The UPS has emerged as an important mechanism by which mitochondrial proteins can be degraded whilst preserving mitochondrial function. Key molecules that regulate mitochondrial membrane dynamics are proteasome substrates, supporting a model of perturbed fission/fusion following 26S proteasomal dysfunction [22], [25]. The accumulation of aberrant mitochondria in 26S proteasome-depleted neurones is also redolent of Parkin-mediated mitophagy, a process that was recently shown to be facilitated by the UPS [25], [72]. Significantly, one study functionally linked the UPS and ALP for mitophagy by showing that 26S proteasome-mediated degradation of outer mitochondrial membrane proteins was essential for mitophagy [38]. In this context our findings are important because 26S proteasomal dysfunction did not lead to activation of the ALP. The mitochondria-containing inclusions in 26S proteasome-depleted neurones may reflect impaired mitophagy. Our study supports a role for the UPS in mitochondrial homeostasis in mammalian neurones *in vivo*.

The molecular events regulating mitochondrial homoeostasis in neurones, including why impairment of the UPS leads to mitochondrial compromise and how this may signal for neuronal death, require further characterization to understand their relevance to neurodegeneration and diseases such as PD. We suggest subtle disturbances in mitochondrial homeostasis through impairment of critical cellular systems such as the UPS with aging may lead to the accumulation of dysfunctional mitochondria and neurodegeneration. Pale bodies contain numerous mitochondria and it is essential to understand the reasons behind their presence in paranuclear inclusions [13]. Interestingly, recent publications suggest α-synuclein may also be directly involved in mitochondrial homeostasis [26]–[28], [73]–[76]. Therefore, mutations or multiplications of *Snca* may act, similarly to products of PD-related genes linked to mitochondria and well-known environmental factors, as well as proteasomal dysfunction, to negatively influence mitochondrial homeostasis and lead to neurodegeneration.

In summary, our data highlight the importance of considering broader mechanistic models, which also have connections to α-synuclein, in events leading to neurodegeneration. For example, one hypothetical model that may explain our findings and connect the elements implicated in PD is failure of mitochondrial homeostasis, with degradative systems (UPS, aggresome, ALP, mitophagy) downstream of several factors known to be involved in maintaining mitochondrial function, including α-synuclein. Proteasomal inhibition and downstream effects on mitochondrial homeostasis may be a critical feature leading to neurodegeneration worthy of further investigation.

## Supporting Information

Figure S1 (A) Double immunofluorescent staining for cytochrome C (mitochondria, green) and LC3 (autophagosomes, red) of sections from control (*Psmc1* ^fl/fl^;*CaMKIIα-Wt*) and 26S proteasome-depleted (*Psmc1* ^fl/fl^;*CaMKIIα-Cre*) mice. Enlarged views of the boxed areas are shown (zoom). Scale bar, 10 µm. (B) LC3-immunopositive inclusions were evident in some nigral (*Psmc1* ^fl/fl^;*TH* ^Wt^;*Snca* ^+/+^) and cortical (*Psmc1* ^fl/fl^;*CaMKIIα-Wt*;*Snca* ^+/+^) neurones, irrespective of the presence or absence of α-synuclein (data not shown). Scale bar, 10 µm.
(TIF)
