# Supplementary figures and images for: Pale Body-Like Inclusion Formation and Neurodegeneration following Depletion of 26S Proteasomes in Mouse Brain Neurones are Independent of α-Synuclein
Source: PLoS One. 2013 Jan 30;8(1):e54711. doi: 10.1371/journal.pone.0054711 (PMC3559752; doi:10.1371/journal.pone.0054711)

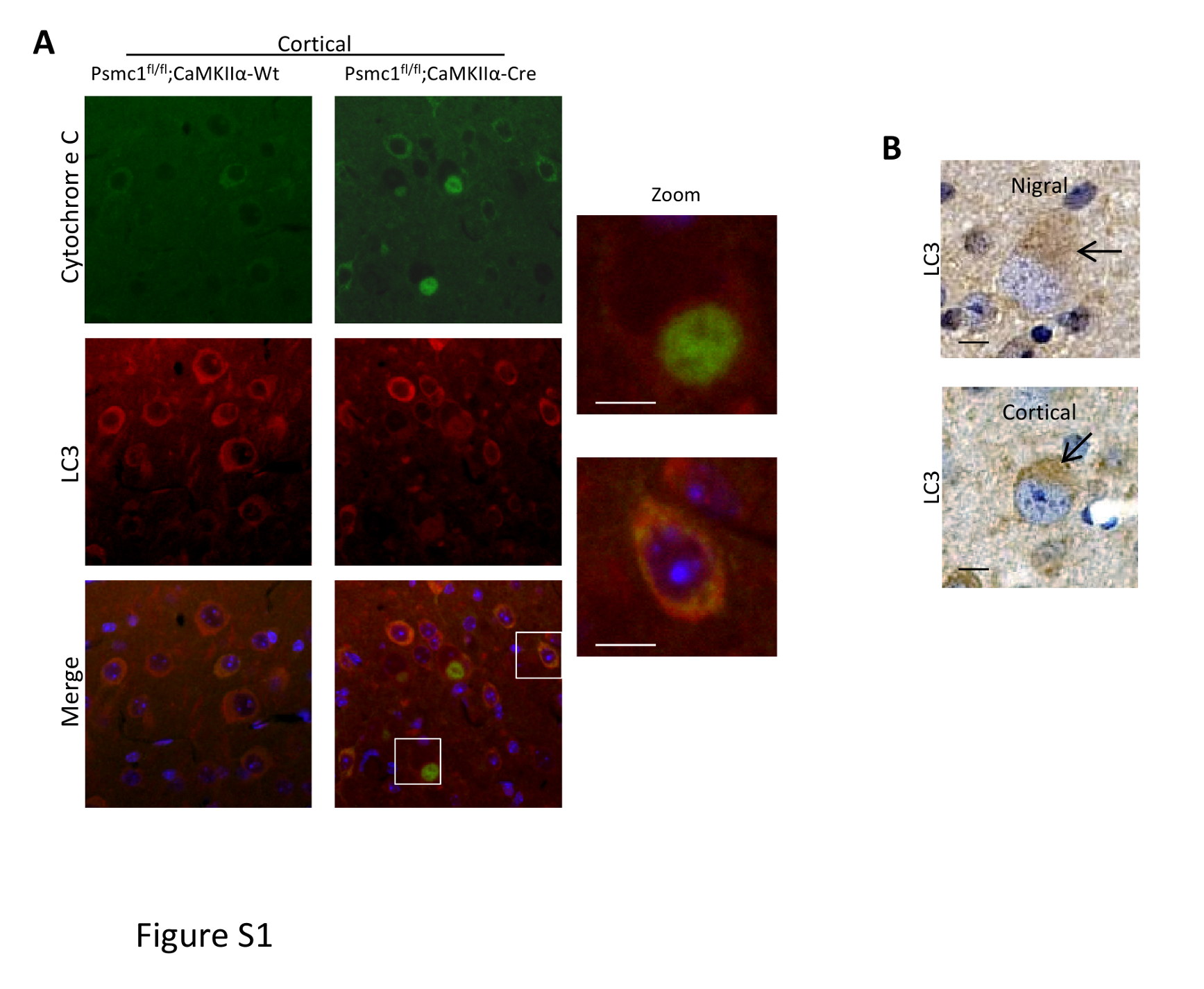

Supplement: Figure S1 — (A) Double immunofluorescent staining for cytochrome C (mitochondria, green) and LC3 (autophagosomes, red) of sections from control (Psmc1 fl/fl;CaMKIIα-Wt) and 26S proteasome-depleted (Psmc1 fl/fl;CaMKIIα-Cre) mice. Enlarged views of the boxed areas are shown (zoom). Scale bar, 10 µm. (B) LC3-immunopositive inclusions were evident in some nigral (Psmc1 fl/fl;TH Wt;Snca +/+) and cortical (Psmc1 fl/fl;CaMKIIα-Wt;Snca +/+) neurones, irrespective of the presence or absence of α-synuclein (data not shown). Scale bar, 10 µm. (TIF) [file pone.0054711.s001.tif]
